# Equality in the distribution of health material and human resources in Guangxi: evidence from Southern China

**DOI:** 10.1186/s13104-017-2760-0

**Published:** 2017-08-29

**Authors:** Jian Sun

**Affiliations:** School of Humanities and Social Science, Guangxi Medical University, 22 Shuang Yong Road, Qing Xiu District, Guangxi Zhuang Autonomous Region, Nanning, 530021 China

**Keywords:** Equality, Health material and human resources, Concentration index, Guangxi

## Abstract

**Objective:**

The aim of this study was to assess the equality in the distribution of health material and human resources in Guangxi, and put forward proposal to improve the equality status of the health material and human resources.

**Results:**

We used concentration index to evaluate the degree of income-related equality of health material and human resources. The concentration index values of the five resources ranged from −0.0847 to 0.1416 from 2011 to 2015. Health institution was concentrated among the poorer populations, while other four resources were concentrated among the richer populations. Overall, the equality status of health institutions, health care beds, health technical personnel, and certified nurses got better from 2011 to 2015. However, the equality status of practicing physicians has got worse since 2014.

**Electronic supplementary material:**

The online version of this article (doi:10.1186/s13104-017-2760-0) contains supplementary material, which is available to authorized users.

## Introduction

Allocating the health material and human resources equitably is one of the foundations of health services development [1]. However, most of Guangxi’s health material and human resources are concentrated among the developed areas, while the remote and developing areas have fewer resources [2]. Inequality in the distribution of health material and human resources seriously hinders the overall improvement of health, which is a concern in the health reform and Healthy Guangxi 2030 strategy.

Evaluating equality in the distribution of health material and human resources in Guangxi is particularly significant for several reasons. On the one hand, Guangxi is an underdeveloped area in China [3]. There exist regional gaps in socio-economic development and health material and human resources allocation within Guangxi [4]. Thus, the equality status in the distribution of health material and human resources is not good [4]. On the other hand, health material and human resources are important economic resources, playing a vital role in improving people’s overall health [5]. The unbalance distribution of health material and human resources hinders the availability of health services, which might result in social conflicts in Guangxi [6].

Studies have showed that the equality status of health material and human resources in Guangxi was poor and needed to be improved. A study in Guangxi found that the health human resources were mainly concentrated among the richer populations, and the equality status remained to be improved [2]. Guo [7] reported that the equality status of health material and human resources allocation in the 14 cities of Guangxi is poor, thus the government should increase the number of health resources and constantly improve the equality status of health material and human resources. Sun [8] concluded that the equality status of health care beds and health technical personnel in Guangxi was much better than that of health institutions and practicing physicians. The aim of this study was to assess the equality in the distribution of health material and human resources in Guangxi, and put forward proposal to improve the equality status of the health material and human resources. The results of this study could provide references for the government to rationalize the allocation of health material and human resources in Guangxi.

## Main text

### Methods

#### Data sources and statistical analysis

Demographic, economic, geographic area, and health material and human resources data was obtained from the Guangxi Statistical Yearbook 2012–2016 [9–13]. Furthermore, Microsoft Excel 2013 was employed to enter data, draw figures and calculate concentration index.

#### Concentration index

Concentration index was adopted to evaluate the degree of income-related equality of health material and human resources. The concentration index is defined as twice the area between the concentration curve and the line of equality (the diagonal line) [14]. The concentration index was calculated as:

$$ {\text{S}} = \frac{1}{2}\sum\limits_{i = 0}^{n - 1} {(Yi + Yi + 1)(Xi + 1 - Xi)} $$

$$ {\text{CI}} = 2\times \left( {0. 5- {\text{S}}} \right), $$

where Y_0_ equals 0 and X_0_ equals 0; Y_i_ is the cumulative proportion of health material and human resources, X_i_ is the cumulative proportion of population, and i is the fractional rank according to per capita gross domestic product beginning with the lowest; CI represents the concentration index [15].

The concentration index ranges between −1 (pro-poor) and +1 (pro-rich); the greater the absolute value of concentration index, the greater the degree of inequalities; a value of zero indicates absolute equality; a negative value indicates a concentration of the resource on the poorer populations; a positive value indicates a concentration of the resource on the richer populations [16–26].

#### Main indicators

Based on a review of related literature [2], we selected the health institutions, health care beds, health technical personnel, practicing physicians, and certified nurses as evaluation indicators. The definitions of the five indicators are shown as following.

1. Health institutions refer to the institutions that have got the legal registration certificates from the health department, such as hospitals, primary health institutions, public health institutions and other health institutions [1].
2. Health care beds refer to the number of beds in health institutions [1].
3. Health technical personnel refers to practicing physicians, certified nurses, pharmacists, radiologists, and other health professionals that work in health institutions [1].
4. Practicing physicians refer to the physicians that have got the legal practicing physicians certificates [1].
5. Certified nurses refer to nurses who have obtained the legal practicing nurses certificates [1].

#### Guangxi Zhuang Autonomous Region

Guangxi Zhuang Autonomous Region is an ethnic minority region in southern China and includes 14 prefecture-level cities. Guangxi has 11 ethnic minority groups, including the Zhuang, Yao, and Miao et al., which accounts for 38% of its population [27]. Guangxi is a less developed province in China, and the per capita gross domestic product of Guangxi was about 36,374 Yuan (approximately $5269) [3].

### Results

#### Basic information of health material and human resources allocation in Guangxi from 2011 to 2015

**Fig. 1:**
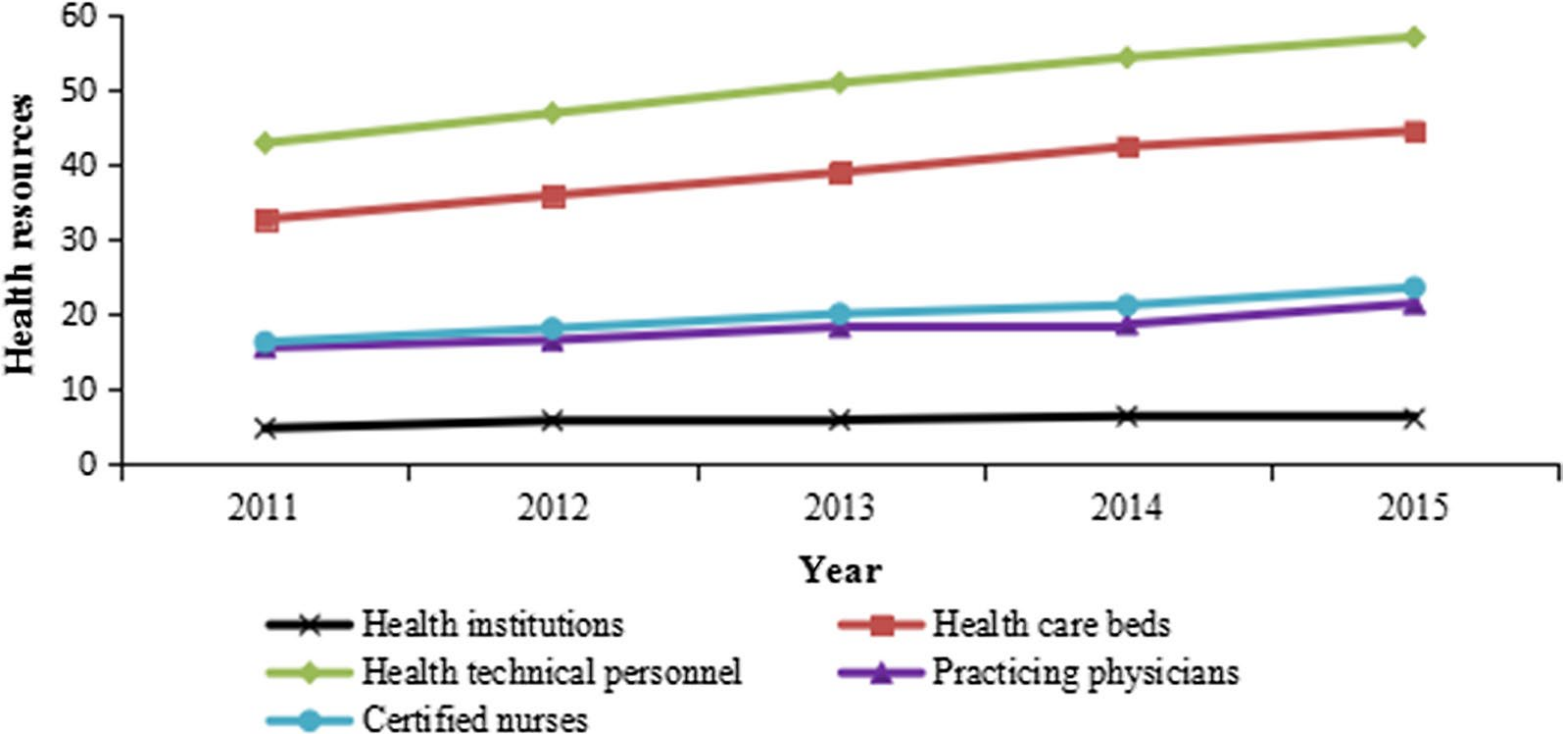
Health material and human resources per 10,000 persons from 2011 to 2015

Table 1 showed the basic information of health material and human resources allocation in Guangxi from 2011 to 2015. The number of the five health material and human resources had been increasing over time. Totally, the number of health institutions, health care beds, health technical personnel, practicing physicians, and certified nurses per 10,000 persons was all increased from 2011 to 2015 (Fig. 1), and they rose 26.45, 36.01, 33.02, 37.50, and 44.85%, respectively. Meanwhile, the physician-nurse ratio was about 1:1.05–1.14.

**Table 1:** Basic information of health material and human resources allocation from 2011 to 2015

Year	Population (10,000 persons)	Health institutions		Health care beds		Health technical personnel		Practicing physicians		Certified nurses		Practicing physicians/certified nurses
		Number	Per 10,000 persons	Number	Per 10,000 persons	Number	Per 10,000 persons	Number	Per 10,000 persons	number	Per 10,000 persons	
2011	4645	22,473	4.84	152,236	32.77	199,457	42.94	72,446	15.60	75,713	16.30	1.05
2012	4682	27,355	5.84	168,244	35.93	219,785	46.94	77,568	16.57	84,897	18.13	1.09
2013	4719	28,071	5.95	184,139	39.02	240,607	50.99	86,540	18.34	94,738	20.08	1.09
2014	4754	30,656	6.45	201,963	42.48	258,618	54.40	88,894	18.70	100,951	21.23	1.14
2015	4796	29,332	6.12	213,748	44.57	273,949	57.12	102,887	21.45	113,236	23.61	1.10

#### Regional distribution of health material and human resources in Guangxi in 2015

In order to make a deeper understanding of health material and human resources distribution in Guangxi, we calculated the number of the resources per 10,000 persons and per square kilometer in the year of 2015.

Table 2 and Additional file 1: Figure S1 showed the regional distribution of health material and human resources per 10, 000 persons in 2015. The number of health institutions, health care beds, health technical personnel, practicing physicians, and certified nurses per 10,000 persons was 6.12, 44.57, 57.12, 21.45, and 23.61, respectively. The number of health institutions per 10,000 persons in Guilin was 32 times more than that in Qinzhou. Compared with the number of health institutions, the degrees of disparity in other four resources were not as great.

**Table 2:** Regional distribution of health material and human resources per 10, 000 persons in 2015

City	Population (10,000 persons)	Health institutions	Health care beds	Health technical personnel	Practicing physicians	Certified nurses
Nanning	698.61	3.95	58.77	82.27	28.87	35.73
Liuzhou	392.27	5.81	55.55	74.03	24.30	32.32
Guilin	496.16	10.70	39.56	60.42	39.61	25.01
Wuzhou	299.94	5.66	37.82	55.58	18.59	23.36
Beihai	162.57	6.47	49.30	55.78	20.16	22.98
Fangchenggang	91.84	6.79	43.06	57.51	19.96	22.54
Qinzhou	320.93	0.33	42.02	50.06	15.51	19.87
Guigang	429.37	9.98	33.50	42.93	13.72	15.96
Yulin	570.72	6.12	39.26	42.72	14.59	16.38
Baise	359.67	7.30	46.01	54.32	16.19	22.12
Hezhou	202.59	5.73	36.55	49.27	15.17	20.08
Hechi	347.68	6.58	44.78	52.34	16.39	21.49
Laibin	218.20	0.98	45.45	43.30	15.72	18.29
Chongzuo	205.45	7.01	40.11	50.58	27.34	21.00
Total/average	4796.00	6.12	44.57	57.12	21.45	23.61

Additional file 2: Table S1 and Additional file 3: Figure S2 presented the regional distribution of health material and human resources per square kilometer in 2015. The number of health institutions, health care beds, health technical personnel, practicing physicians, and certified nurses per square kilometer was 0.12, 0.90, 1.16, 0.43, and 0.48, respectively. The number of health institutions per square kilometer in Guigang was 40 times more than that in Qinzhou. Among the five resources, the number of health technical personnel per square kilometer in the 14 cities showed the smallest gap.

#### Analysis of concentration index

The concentration index values of health material and human resources by population from 2011 to 2015 were shown in Additional file 4: Table S2. The concentration index values of the five resources ranged from −0.0847 to 0.1416 at the same time. The concentration index values of health institutions were negative, indicating this resource was concentrated among the poorer populations. In contrast, the concentration index values of other four resources were positive, indicating these resources were concentrated among the richer populations. The absolute values of concentration index of health technical personnel, practicing physicians, and certified nurses were significantly higher than those of health institutions and health care beds, which suggests that the equality status of material resources was better than that of human resources.

Additional file 5: Figure S3 presented the concentration index values of health material and human resources by population from 2011 to 2015. In the meanwhile, the concentration index values of health institutions showed an overall upward trend, while the concentration index values of health care beds, health technical personnel, and certified nurses showed an overall downward trend, indicating the absolute values of concentration index of the four resources showed an overall downward trend. That is to say, the equality status of the four resources got better. Conversely, the absolute values of concentration index of practicing physicians showed an overall upward trend, indicating the equality status of the resource got worse.

## Discussion

As mentioned above, totally, the equality status of health institutions, health care beds, health technical personnel, and certified nurses got better. This situation was consistent with other studies [8, 28]. One reason leading to this trend could be the health reform in 2009. The central government of China has invested 127 million dollars to develop health material and human resources in health institutions to narrow the gaps in health services among people in different regions since 2009, which greatly improved the equality status of health material and human resources in different regions [29]. However, the equality status of practicing physicians has got worse since 2014. In addition, the concentration index values of practicing physicians were the highest among the five resources in the year of 2015, which indicates that the equality status was the worst. The most potential explanation for this result was the gaps of economics in different regions of Guangxi. Economically developed areas, such as Nanning, Guilin, and Liuzhou, can provide higher salary and better career development opportunities than the remote and underdeveloped areas [8]. Thus, practicing physicians prefer to work in developed areas, whilst the remote and underdeveloped areas, such as Yulin, Guigang, and Laibin, fail to attract adequate practicing physicians [2]. Consequently, the government should pay attention to the equality status of practicing physicians, and formulate preferential policies to encourage practicing physicians to work in remote and underdeveloped areas of Guangxi, and formulate policies to set up the rational flow mechanism of practicing physicians to improve the equality status of practicing physicians [30]. In addition, the health institutions are supposed to introduce adequate practicing physicians in remote and economically underdeveloped areas by giving extra subsidies and other preferential policies to ameliorate the inequality status of practicing physicians [31]. Moreover, the medical universities in Guangxi need to enroll more clinical medical students and encourage them to work in remote and underdeveloped areas of Guangxi by giving some prizes [32].

In conclusion, this study provides important suggestive evidence for the equality in the distribution of health material and human resources in Guangxi by elucidating the changing trends of concentration index from 2011 to 2015. This study proves that concentration index was suitable to evaluate the equality of health material and human resources in Guangxi, and it is first time to find that health institutions in Guangxi were concentrated among the poorer populations.

### Limitations

There are several limitations in this study. On the one hand, as the Guangxi Statistical Yearbook does not include the health investment of each city, we do not have the relevant data to assess the equality status of health investment. On the other hand, gender equality is a significant issue in health material and human resources allocation, but due to the limitation of the data, we are not able to evaluate the gender equality status of the health material and human resources.

## Additional files

**Additional file 1: Figure S1.** Regional distribution of health material and human resources per 10, 000 persons in 2015.

**Additional file 2: Table S1.** Regional distribution of health material and human resources per square kilometer in 2015.

**Additional file 3: Figure S2.** Regional distribution of health material and human resources per square kilometer in 2015.

**Additional file 4: Table S2.** Concentration index values of health material and human resources by population from 2011 to 2015.

**Additional file 5: Figure S3.** Concentration index values of health material and human resources by population from 2011 to 2015.
