# Supplementary figures and images for: Equality in the distribution of health material and human resources in Guangxi: evidence from Southern China
Source: BMC Res Notes. 2017 Aug 29;10:429. doi: 10.1186/s13104-017-2760-0 (PMC5576300; doi:10.1186/s13104-017-2760-0)

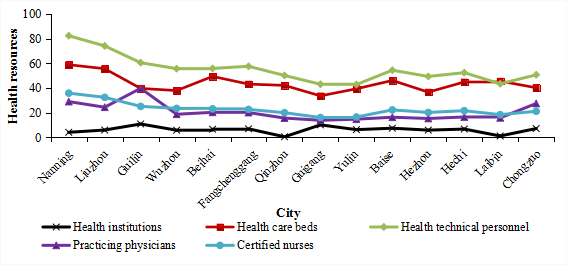

Supplement: Supplementary file 1 — Additional file 1: Figure S1. Regional distribution of health material and human resources per 10, 000 persons in 2015. [file 13104_2017_2760_MOESM1_ESM.doc]

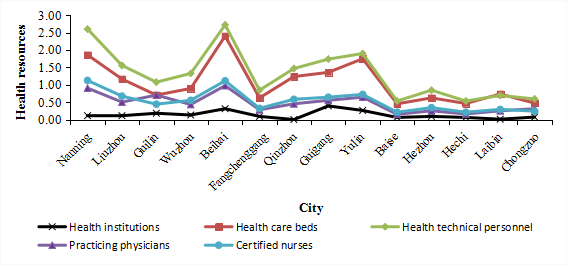

Supplement: Supplementary file 3 — Additional file 3: Figure S2. Regional distribution of health material and human resources per square kilometer in 2015. [file 13104_2017_2760_MOESM3_ESM.doc]

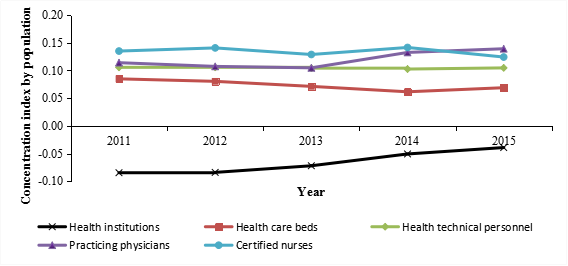

Supplement: Supplementary file 5 — Additional file 5: Figure S3. Concentration index values of health material and human resources by population from 2011 to 2015. [file 13104_2017_2760_MOESM5_ESM.doc]
