# Supplementary material for: Equality in the distribution of health material and human resources in Guangxi: evidence from Southern China
Source: BMC Res Notes. 2017 Aug 29;10:429. doi: 10.1186/s13104-017-2760-0 (PMC5576300; doi:10.1186/s13104-017-2760-0)
Supplement: Supplementary file 2 — Additional file 2: Table S1. Regional distribution of health material and human resources per square kilometer in 2015. [file 13104_2017_2760_MOESM2_ESM.doc]

**Additional file 2: Table S1 Regional distribution of health material and human resources per square kilometer** in 2015

| City | Area  (square kilometer) | Health institutions | Health care beds | Health technical personnel | Practicing physicians | Certified nurses |
| --- | --- | --- | --- | --- | --- | --- |
| Nanning | 22,099 | 0.12 | 1.86 | 2.60 | 0.91 | 1.13 |
| Liuzhou | 18,597 | 0.12 | 1.17 | 1.56 | 0.51 | 0.68 |
| Guilin | 27,809 | 0.19 | 0.71 | 1.08 | 0.71 | 0.45 |
| Wuzhou | 12,572 | 0.14 | 0.90 | 1.33 | 0.44 | 0.56 |
| Beihai | 3,337 | 0.32 | 2.40 | 2.72 | 0.98 | 1.12 |
| Fangchenggang | 6,238 | 0.10 | 0.63 | 0.85 | 0.29 | 0.33 |
| Qinzhou | 10,895 | 0.01 | 1.24 | 1.47 | 0.46 | 0.59 |
| Guigang | 10,602 | 0.40 | 1.36 | 1.74 | 0.56 | 0.65 |
| Yulin | 12,838 | 0.27 | 1.75 | 1.90 | 0.65 | 0.73 |
| Baise | 36,201 | 0.07 | 0.46 | 0.54 | 0.16 | 0.22 |
| Hezhou | 11,753 | 0.10 | 0.63 | 0.85 | 0.26 | 0.35 |
| Hechi | 33,476 | 0.07 | 0.47 | 0.54 | 0.17 | 0.22 |
| Laibin | 13,411 | 0.02 | 0.74 | 0.70 | 0.26 | 0.30 |
| Chongzuo | 17,332 | 0.08 | 0.48 | 0.60 | 0.32 | 0.25 |
| Total/average | 237,160 | 0.12 | 0.90 | 1.16 | 0.43 | 0.48 |
