# Supplementary material for: Equality in the distribution of health material and human resources in Guangxi: evidence from Southern China
Source: BMC Res Notes. 2017 Aug 29;10:429. doi: 10.1186/s13104-017-2760-0 (PMC5576300; doi:10.1186/s13104-017-2760-0)
Supplement: Supplementary file 4 — Additional file 4: Table S2. Concentration index values of health material and human resources by population from 2011 to 2015. [file 13104_2017_2760_MOESM4_ESM.doc]

Additional file 4: Table S2 Concentration index values of health material and human resources by population from 2011 to 2015

| Year | Health institutions | Health care beds | Health technical personnel | Practicing physicians | Certified nurses |
| --- | --- | --- | --- | --- | --- |
| 2011 | -0.0847 | 0.0849 | 0.1057 | 0.1141 | 0.1351 |
| 2012 | -0.0841 | 0.0804 | 0.1057 | 0.1074 | 0.1408 |
| 2013 | -0.0721 | 0.0713 | 0.1042 | 0.1048 | 0.1289 |
| 2014 | -0.0508 | 0.0615 | 0.1026 | 0.1326 | 0.1416 |
| 2015 | -0.0392 | 0.0689 | 0.1047 | 0.1393 | 0.1243 |
